# Retinal imaging study diagnoses in COVID-19: a case report

**DOI:** 10.1186/s13256-020-02620-5

**Published:** 2021-01-15

**Authors:** José M. Ortiz-Egea, Jorge Ruiz-Medrano, José M. Ruiz-Moreno

**Affiliations:** 1 Department of Ophthalmology, Castilla La Mancha University, Albacete, Spain; 2 Puerta de Hierro-Majadahonda University Hospital, Madrid, Spain; 3 Red Temática de Investigación Cooperativa en Salud: “Prevención, detección precoz, y tratamiento de la patología ocular prevalente, degenerativa y crónica”. RD16/0008/0021, Spanish Ministry of Health, Instituto de Salud Carlos III, Madrid, Spain

**Keywords:** OCT, COVID-19, SARS-CoV-2, Ophthalmology, Retina

## Abstract

**Background:**

Hyperreflective lesions at the level of ganglion cell (GCL) and inner plexiform retinal layers (IPL) by optical coherence tomography (OCT) and cotton wool spots in the examination of the eye fundus have recently been described as findings in patients with COVID-19 infection.

**Case report:**

We report the case of a 42-year-old healthy Caucasian male anesthetist who had treated COVID-19 patients during the previous 5 weeks and suddenly presented with a temporal relative scotoma in his left eye. Best-corrected visual acuity was 20/20 for the left eye, and no discromatopsy or afferent pupillary defect was present. Visual field test was performed, with no significant findings associated with the focal loss of sensitivity described by the patient. The anterior segment was unremarkable on slit lamp examination in both eyes. Fundus examination of the left eye showed no significant findings. A placoid, hyperreflective band at the level of the GCL and IPL was visible in OCT which spared the outer retina, at the time of diagnosis and 1 month later. An oropharyngeal swab test was performed for severe acute respiratory syndrome coronavirus 2 (SARS-CoV-2) ribonucleic acid (RNA), immunoglobulin G (IgG) and immunoglobulin M (IgM) enzyme-linked immunosorbent assay (ELISA) determination. Real-time reverse-transcriptase polymerase chain reaction (RT-PCR) was negative. ELISA testing and a third rapid antibody detection test performed 7 days after the onset of symptoms were positive.

**Conclusions:**

Ocular signs and symptoms in COVID-19 cases are rarely reported, but may be underestimated, especially those that affect the retina and occur in asymptomatic or paucisymptomatic cases. We present a case of COVID-19 diagnosis based on retinal ophthalmic examination.

## Background

Coronavirus disease 2019 (COVID-19) is caused by severe acute respiratory syndrome coronavirus 2 (SARS-CoV-2). In humans, diseases of the coronavirus family range from the mild common cold to more severe diseases such as Middle East respiratory syndrome (MERS) and SARS [1].

COVID-19 can cause pathological ophthalmologic involvement, including conjunctivitis, chemosis, hyperemia, epiphora, secretion [2], photophobia, dry eye [3], neuro-ophthalmic manifestations such as optic neuritis, cranial nerve palsies, nystagmus or visual field defects [4], and descriptions of retinal aggression [5, 6].

Optical coherence tomography (OCT) has proved to be a useful tool for the *in vivo* study of the retina, where hyperreflective structures range from normal retina (nerve, fiber layer, inner and outer plexiform layers), nerve fiber layer myelination or vessels, to lesions such as hard exudates, haemorrhages, fibrosis, or focal inflammation, among others. Some of those imply the appearance of a shadow below said structures that may lead to interpretation errors [7].

## Case presentation

We present the case of a 42-year-old, healthy Caucasian male anesthetist who had been working with COVID-19 patients during the 5 weeks prior to onset, who presented with a sudden temporal relative scotoma in the left eye. The patient had no previous retinal disease or systemic disease with retinal compromise. Best-corrected visual acuity was 20/20 for the left eye, and no discromatopsy or afferent pupillary defect was present. A visual field test (SITA Fast 30-2) was performed, with no significant findings associated with a focal loss of sensitivity described by the patient. The anterior segment and fundus examination were unremarkable in both eyes.

**Fig. 1:**
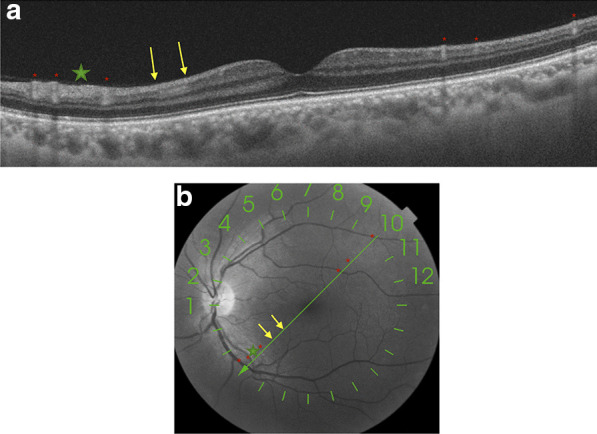
**a** and **b** Swept-source optical coherence tomography (SS-OCT, Topcon Co., Tokyo, Japan) showed a hyperreflective band (yellow arrows) at the level of the ganglion cell and inner plexiform retinal layers, which spared the outer retina. Green line where the B-scan of the OCT was acquired superposed automatically by the acquisition instrument on an *en face* infrared fundus image, where there are signs of arterial and venous vessel reflexes (red asterisks) and nerve fiber layer hyperreflectivity (green star)

Swept-source optical coherence tomography (SS-OCT, Topcon Co., Tokyo, Japan) showed a hyperreflective band at the level of ganglion cell and inner plexiform layers, which spared the outer retina (Fig. 1a, b). Multimodal imaging showed neither hypo- nor hyper-autofluorescence in the area. Fluorescein angiography showed no areas of leakage or vascular exudation in early or late phases.

The patient did not report respiratory symptoms, fever or any other clinical symptoms typically described in COVID-19 cases. Thoracic computed tomography imaging did not show lesions compatible with those described in COVID-19 cases with respiratory involvement. Blood tests performed were normal, with no signs of coagulopathy alterations. The patient had normal blood pressure values.

After identifying the aforementioned retinal lesions and considering the patient’s high-risk profession with regard to COVID-19 exposure, a pharyngeal swab test for SARS-CoV-2 ribonucleic acid (RNA) and enzyme-linked immunosorbent assay (ELISA) determination of immunoglobulin G (IgG) and immunoglobulin M (IgM) were requested. At that time, the patient remembers that he had limited ageusia for several days 3 weeks before the onset of the scotoma. Real-time reverse-transcriptase polymerase chain reaction (RT-PCR) was negative. ELISA testing and a third rapid antibody detection test performed 7 days after the onset of symptoms were positive.

**Fig. 2:**
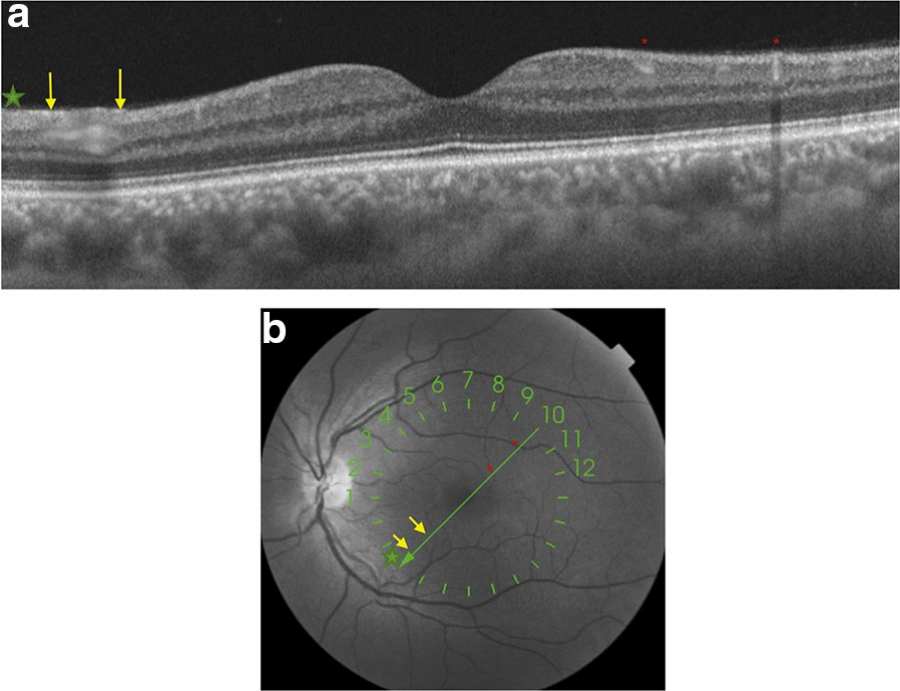
**a** and **b** One month later, SS-OCT follow-up shows a more prominent hyperreflective band at the level of ganglion cell and inner plexiform retinal layers (yellow arrows). An *en face* infrared fundus image with a green line where the OCT B-scan was acquired automatically overlaid by the acquisition instrument. On the left, the hyperreflective signal corresponds to the layer of nerve fibers (green star) and reflex vessels (red asterisks)

In the subsequent follow-up of the patient 30 days after the start of the scotoma perception, he continued to describe it. Retinal imaging study showed the same hyperreflective lesions observed in SS-OCT, with even greater intensity (Fig. 2a, b), and there were no arteries or veins in the inner layers of the retina on this B-scan that could cause a hyperreflective shadow.

## Discussion and conclusions

Back in 2013, Sarraf *et al.* were the first to describe the presence of multiple or isolated band-shaped, focal or diffuse hyperreflective lesions visible at the level of the internal nuclear layer in patients who presented with acute onset of a negative scotoma, which they called paracentral acute medial maculopathy (PAMM). PAMM is a spectral-domain OCT (SD-OCT) finding interpreted as a possible more superficial variant of acute macular neuroretinopathy [8].

Its etiology is unknown, although the most commonly supported hypothesis is based on a vascular origin. A localized retinal capillary ischemia at the level of the intermediate plexus has been proposed as the underlying mechanism for the development of these lesions.

As Chen *et al.* describe, retinal vascular associations leading to retinal vasculopathy and PAMM include eye compression injuries causing global ocular ischemia, sickle cell crisis, Purtscher's retinopathy, inflammatory occlusive retinal vasculitis, pandemic influenza A (H1N1) vaccines reaction, hypertensive retinopathy, migraine disorder and upper respiratory infection [9]. None of these clinical findings was present in our patient.

Early clinical evidence suggests that cases of COVID-19 are frequently characterized by hyperinflammation, renin–angiotensin–aldosterone system imbalance, and a particular form of vasculopathy, thrombotic microangiopathy, and intravascular coagulopathy. There are no conclusive studies in paucisymptomatic or subclinical cases [10].

To date, there is very limited evidence of the correlation between COVID-19 and the appearance of retinal lesions, presumably because there is wide clinical variation in the presentation and severity of the disease, which may induce the appearance of different morphological patterns of retinal involvement. Marinho *et al.* [5], for instance, describe the presence of hyperreflective lesions at the level of ganglion cell and inner plexiform layers more prominently at the papillomacular bundle, but we must be extremely careful with these findings because, as Vavvas *et al.* [11] point out, OCT hyperreflective bands in the inner retina and/or ganglion cell layer can be confused with normal inner retinal vessels. Landecho *et al*. [6] recently described cotton wool spots in the retinal fundoscopic examination of the eye and corresponding B-scan OCT showing inflammation of the nerve fiber layer of the retina in 6 of 24 asymptomatic subjects 14 days after discharge from the hospital for treatment for bilateral COVID-19 pneumonia. For this reason, we consider the study with multimodal imaging to be important, agreeing with these authors that we must check at least the near-infrared reflectance record to confirm that the hyperreflective bands do not represent normal vessels (Figs. 1 and 2).

Vascular occlusions described in COVID-19 cases might also be the cause of these retinal findings [10] or may be associated with the neurological manifestations described in animal studies and in COVID-19 neurological events [12–14]. It is possible that these vascular occlusions affect the retinal circulation in its superficial and deep plexuses, generating these areas of hyperreflective signals in the inner layers of the retina. Therefore, and exclusively from an ophthalmological point of view, given the possible implications, COVID-19 infection should be excluded by all available means in cases where these hyperreflective lesions occur at the level of ganglion cell and internal plexiform layer OCT imaging without overlying vessels (infrared fundus imaging helps), to facilitate timely diagnosis and intervention.

This case, and the papers presented by other authors [5, 6, 11], support the hypothesis that these retinal OCT findings should be considered another sign of COVID-19 disease, hence the importance of retinal imaging study in these patients. Furthermore, to the best of our knowledge, ours is a case of COVID-19 diagnosed through an imaging study of the retina.

## Data Availability

Not applicable.

## Abbreviations

COVID-19: Coronavirus disease 2019
GCL: Ganglion cell layer
IPL: Inner plexiform retinal layer
OCT: Optical coherence tomography
OS: Left eye
PAMM: Paracentral acute medial maculopathy
RT-PCR: Real-time reverse-transcriptase polymerase chain reaction
SARS-CoV-2: Severe acute respiratory syndrome coronavirus 2
SD-OCT: Spectral-domain optical coherence tomography
SS-OCT: Swept-source optical coherence tomography
